# Electrosynthesis of Aromatic Poly(amide-amine) Films from Triphenylamine-Based Electroactive Compounds for Electrochromic Applications

**DOI:** 10.3390/polym9120708

**Published:** 2017-12-13

**Authors:** Sheng-Huei Hsiao, Hsing-Yi Lu

**Affiliations:** Department of Chemical Engineering and Biotechnology, National Taipei University of Technology, No. 1, Sec. 3, Chunghsiao East Rd., Taipei 10608, Taiwan; shinhwa904@gmail.com

**Keywords:** triphenylamine, carbazole, electrochemistry, electrochemical polymerization, electrochromism

## Abstract

Two electropolymerizable monomers with a methoxytriphenylamine core linked via amide groups to two triphenylamine (TPA) or *N*-phenylcarbazole (NPC) terminal groups, namely 4,4′-bis(4-diphenylaminobenzamido)-4′′-methoxytriphenylamine (MeOTPA-(TPA)_2_) and 4,4′-bis(4-(carbazol-9-yl)benzamido)-4′′-methoxytriphenylamine (MeOTPA-(NPC)_2_), were synthesized and characterized by FTIR and ^1^H NMR spectroscopy, mass spectrometry, and cyclic voltammetry. The electrochemical polymerization reactions of these MeOTPA-cored monomers over indium tin oxide (ITO) electrode allow the generation of electroactive poly(amide-amine) films. The electro-generated polymer films exhibited reversible redox processes and multi-colored electrochromic behaviors upon electro-oxidation, together with moderate coloration efficiency and cycling stability. The optical density changes (Δ*OD*) were observed in the range of 0.18–0.68 at specific absorption maxima, with the calculated coloration efficiencies of 42–123 cm^2^/C. Single-layer electrochromic devices using the electrodeposited polymer films as active layers were fabricated for the preliminary investigation of their electrochromic applications.

## 1. Introduction

Electrochromism refers to the alternation of optical absorption or color of an electroactive species by electrochemically induced redox reactions [1]. Materials capable of such color switching have found use in many applications such as electronic papers [2,3], automatic-dimming mirrors, smart windows [4], adaptive camouflage [5,6], eyewear [7,8], and energy storage devices [9,10]. A number of organic and inorganic materials have been used to construct electrochromic devices, such as transition metal oxides, inorganic coordination complexes, organic dyes and polymers, and organic-metallic hybrid polymers [11,12,13,14,15]. Among the different types of electrochromic materials, organic polymers attract much interest because of several advantages such as mechanical flexibility, enhanced processability, easy color tuning, rapid switching and high coloration efficiency [16,17,18,19,20].

Triphenylamine (TPA) is a promising core unit for opto- and electro-active materials because of the facile formation of radical cation and its charge transport ability. Many triphenylamine and triarylamine starbursts, dendrimers, and polymers have been used as electron-blocking and hole-transporting layers in organic electronic devices such as organic light-emitting diodes (OLEDs), organic field-effect transistors (OFETs), and dye-sensitized solar cells [21,22,23,24,25]. Since 2005, Liou’s and our groups have extensively studied non-conjugated triarylamine-based polymers for electrochromic applications [26,27,28,29,30,31,32,33,34]. We prepared and characterized various structurally different polyimides and polyamides with triarylamine units and aromatic or non-aromatic spacers in the polymer backbone. The obtained polymers are generally soluble in most of the polar aprotic solvents allowing preparing amorphous thin films via solution casting. In general, these films show strong coloration change upon oxidation and exhibit high optical contrast and cycling stability. The introduction of electron-donating substituents such as methoxy groups into the electrochromic unit in the reactive sites could decrease the oxidation potential and allow the formation of more stable triarylamine radical cations [35]. Thus, a further improvement on the electrochromic performance of the polymers could be obtained using 4,4′-diamino-4′′-methoxytriphenylamine (MeOTPA-(NH_2_)_2_) as a functional diamine monomer [35].

Unsubstituted TPA can be readily electrochemically oxidized to a TPA radical cation. The formed radical cation dimerizes and upon abstraction of two protons yields tetraphenylbenzidine (TPB) [36,37]. The electrochemical coupling reaction of TPA has been used as a convenient and effective method to prepare electroactive polymers for optoelectronic applications [38,39,40,41,42,43]. Systematic studies on the electrochemical oxidative coupling of carbazole have been reported by Ambrose and Nelson [44,45]. By applying oxidative potential, carbazole radical cations are generated that undergo coupling to 3,3′-biscarbazole. Many redox-active and electrochromic polymers have been synthesized via the cabazole-based oxidative coupling polymerization [46,47,48,49,50,51,52]. Additionally, several functional, microporous thin films with high surface areas have been produced by this approach [53]. Electrochemical polymerization allows for simultaneous polymer synthesis and film deposition in short times and under mild and metal- or catalyst-free conditions. In this work, two novel electropolymerizable monomers MeOTPA-(TPA)_2_ and MeOTPA-(NPC)_2_ were synthesized from the condensation reactions of 4,4′-diamino-4′′-methoxytriphenylamine (MeOTPA-(NH_2_)_2_) with 4-carboxytriphenylamine (TPA-COOH) and *N*-(4-carboxyphenyl)carbazole (NPC-COOH), respectively. Electroactive and electrochromic poly(amide-amine) films could be easily built-up on the electrode surface via TPA- or carbazole-based electrochemical oxidative coupling. In comparison with other monomers without redox-active centers [54], the present MeOTPA-centered monomers would result in poly(amide-amine)s with an additional, low-oxidation-potential MeOTPA redox center. Thus, the prepared electroactive polymer films are expected to exhibit lowered onset operating voltages when they are used as active layers in electrochromic devices. Better coloration efficiency and multiple electrochromic switching are also anticipated.

## 2. Results and Discussion

### 2.1. Monomer Synthesis

**Figure 1:**
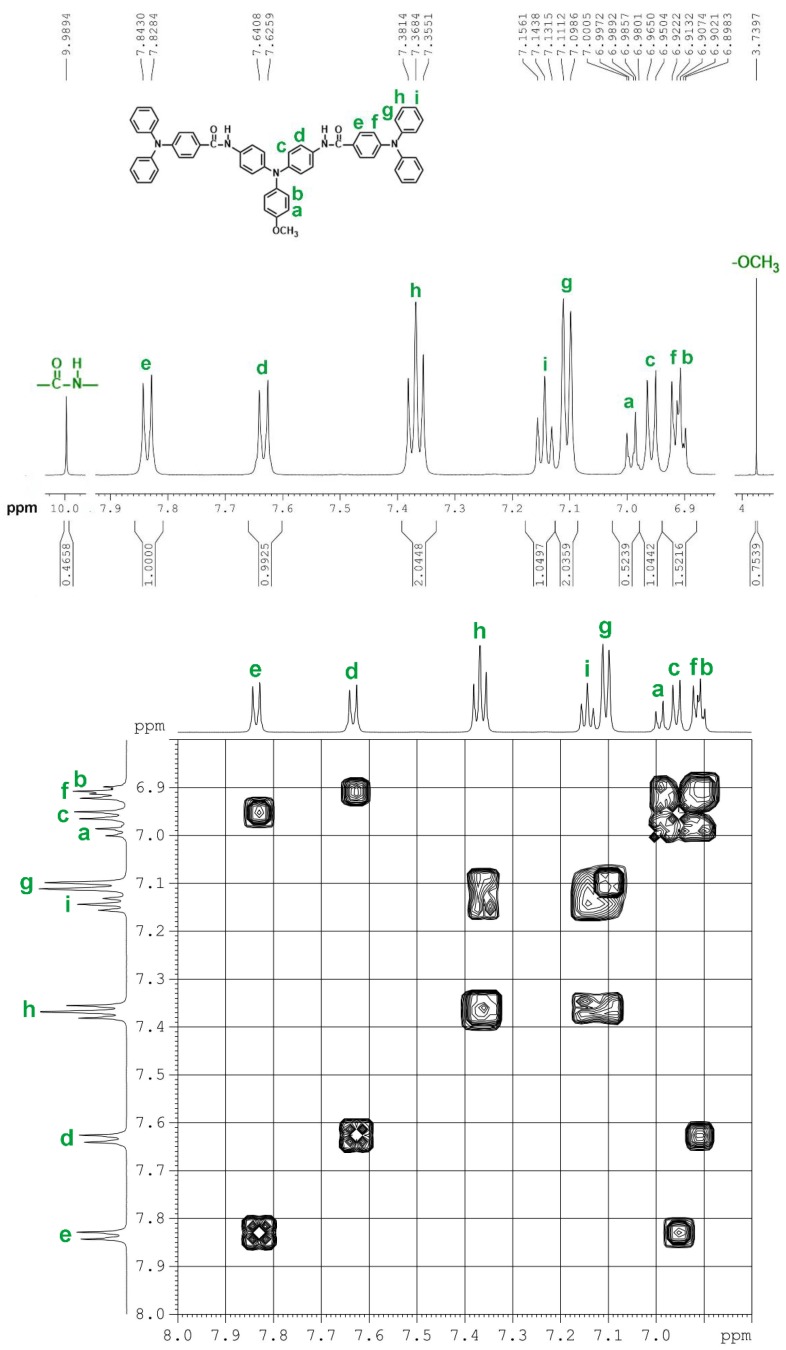
^1^H NMR and H–H correlation spectroscopy (COSY) spectra of MeOTPA-(TPA)_2_ in dimethyl sulfoxide (DMSO)-*d*_6_.

**Figure 2:**
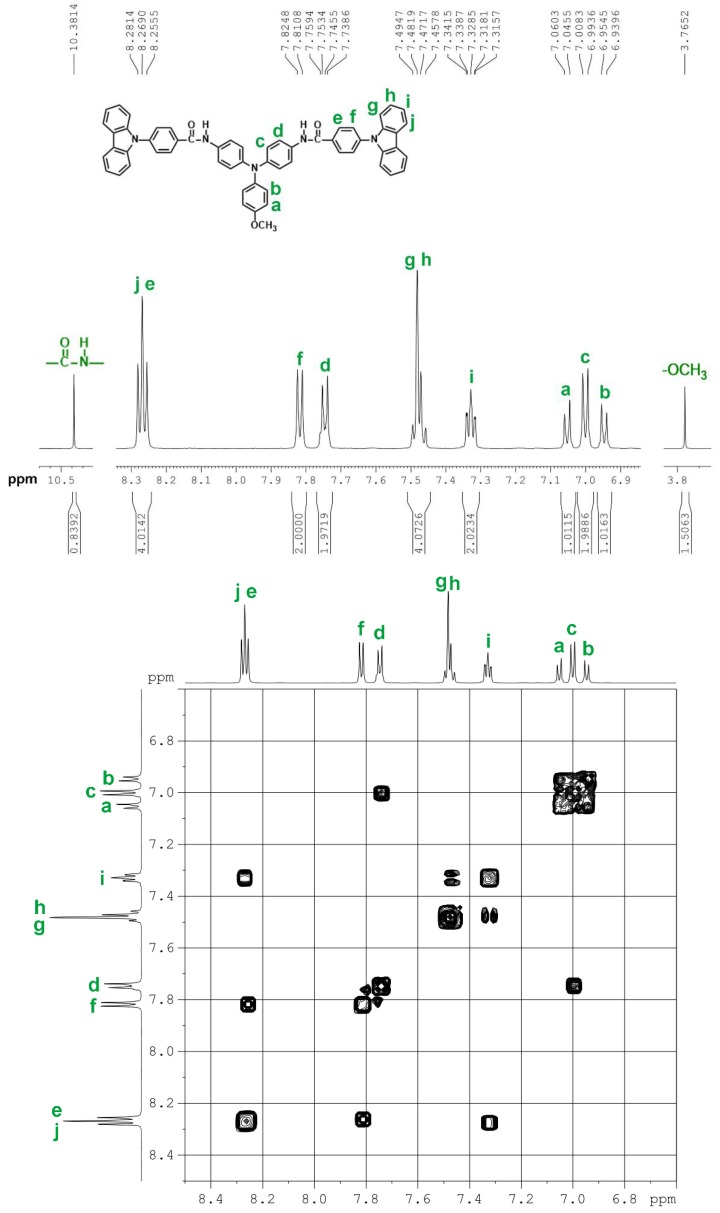
^1^H NMR and H–H COSY spectra of MeOTPA-(NPC)_2_ in DMSO-*d*_6_.

**Scheme 1:**
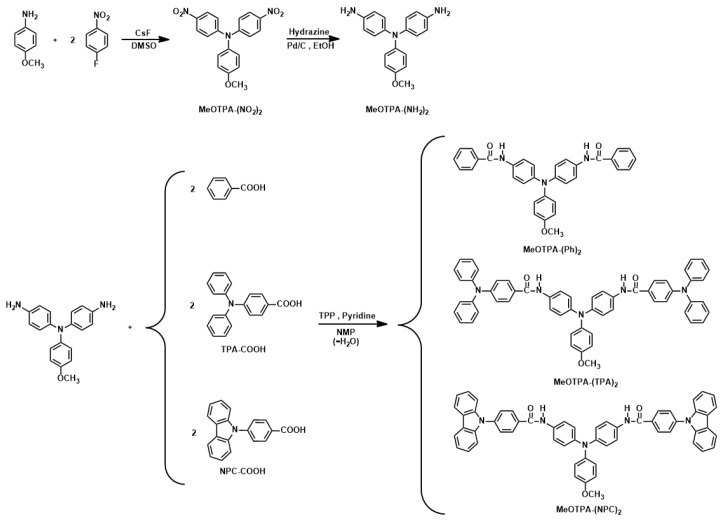
Synthetic route of methoxytriphenylamine (MeOTPA)-cored model compound and monomers.

The MeOTPA-cored diamide monomers MeOTPA-(TPA)_2_ and MeOTPA-(NPC)_2_ and model compound MeOTPA-(Ph)_2_ were prepared by condensation of MeOTPA-(NH_2_)_2_ with 4-carboxytriphenylamine, *N*-(4-carboxyphenyl)carbazole, and benzoic acid, respectively, using triphenyl phosphite (TPP) and pyridine as condensing agents. Synthetic details and characterization data are included in the Supplementary Materials. The synthetic route is shown in Scheme 1, and all the target compounds were characterized by FTIR and ^1^H NMR spectroscopy. Figure S1 (Supplementary Materials) illustrates FT-IR spectra of all the synthesized compounds. The nitro groups of MeOTPA-(NO_2_)_2_ show the characteristic absorptions at 1575 and 1318 cm^−1^ (–NO_2_ asymmetric and symmetric stretching). After reduction, the characteristic absorptions of nitro group disappear and MeOTPA-(NH_2_)_2_ shows the typical –NH_2_ stretching absorption pair at 3334 and 3216 cm^−1^. All of the synthesized compounds MeOTPA-(Ph)_2_, MeOTPA-(TPA)_2_ and MeOTPA-(NPC)_2_ show the characteristic amide absorption bands at around 3267–3279 cm^−1^ (N–H stretching) and 1635–1650 cm^−1^ (amide C=O stretching). The ^1^H and H–H correlation spectroscopy (COSY) NMR spectra of MeOTPA-(TPA)_2_, MeOTPA-(NPC)_2_ and MeOTPA-(Ph)_2_ illustrated in Figure 1, Figure 2 and Figure S2 (Supplementary Materials) agree well with the proposed molecular structures. In addition, mass spectroscopy results of monomers MeOTPA-(TPA)_2_ and MeOTPA-(NPC)_2_ are consistent with their calculated values (Figure S3, Supplementary Materials).

### 2.2. Electrochemical and Spectroelectrochemical Properties of Model Compound

**Figure 3:**
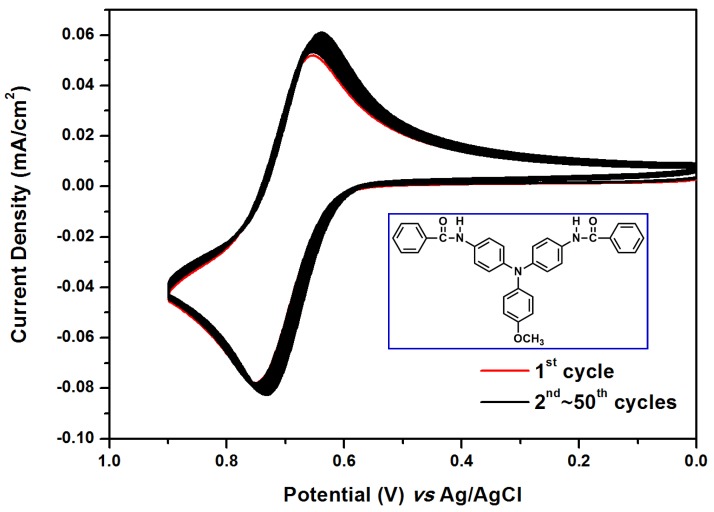
Repetitive cyclic voltammograms (50 cycles) of 2 × 10^−4^ M model compound MeOTPA-(Ph)_2_ in 0.1 M Bu_4_NClO_4_/CH_2_Cl_2_ solution at a scan rate of 50 mV·s^−1^.

**Figure 4:**
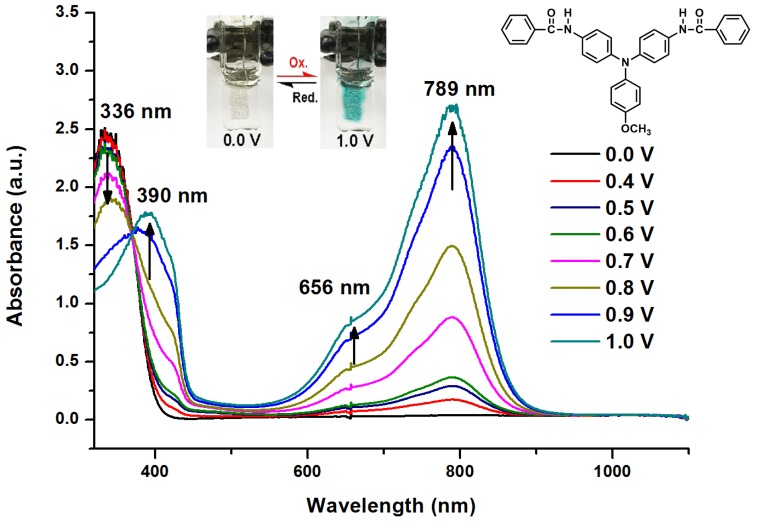
Spectroelectrochemistry of 1 × 10^−3^ M model compound MeOTPA-(Ph)_2_ in 0.1 M Bu_4_NClO_4_/CH_2_Cl_2_.

The electrochemical behavior of a solution 2 × 10^−4^ M model compound (MeO)_2_TPPA-(Ph)_2_ in 0.1 M Bu_4_NClO_4_/CH_2_Cl_2_ electrolyte was examined by cyclic voltammetry (CV) at 50 mV·s^−1^ scan rate. As shown in Figure 3, MeOTPA-(Ph)_2_ showed one pair of reversible redox waves with an oxidation peak at around 0.75 V (*E*_1/2_ = 0.71 V) in the first CV scan. The reversible wave can be attributed to the oxidation process occurring in the MeOTPA core. This model compound showed very stable electrochemical oxidation processes in the first 50 repetitive CV scans between 0 and 0.9 V. Figure 4 shows the optical absorption and color changes of MeOTPA-(Ph)_2_ on a Pt grid in an OTTLE cell. In the neutral form, the compound exhibited a strong absorption at λ_max_ = 336 nm. When the applied potential was gradually raised from 0 to 1.0 V, the absorbance at 336 nm slightly decreased and new bands appeared at 390, 656 and 789 nm. Meanwhile, the color changed to turquoise. The spectral and color changes are apparently attributed to the formation of cationic radical species of this compound caused by oxidation of the MeOTPA core.

### 2.3. Electrochemical Polymerization of Monomers

**Figure 5:**
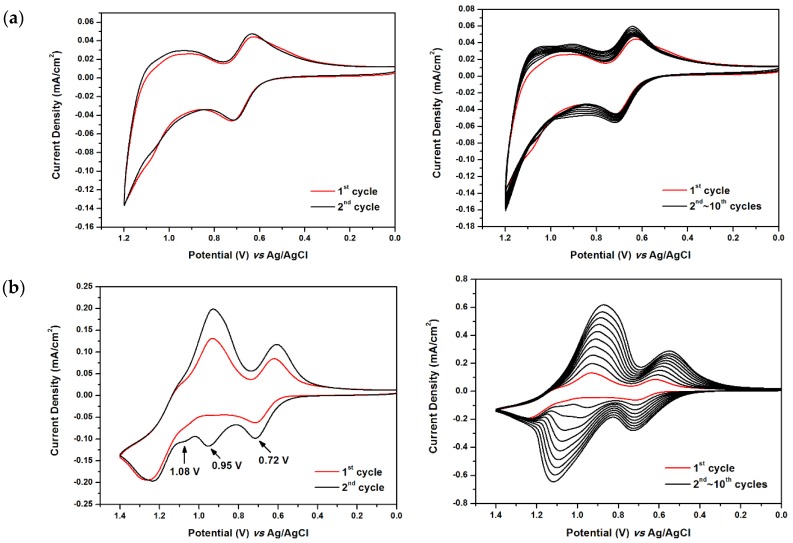
Repetitive cyclic voltammograms of 2 × 10^−4^ M MeOTPA-(TPA)_2_ in 0.1 M Bu_4_NClO_4_/CH_2_Cl_2_ solution in the potential range of (**a**) 0–1.2 V and (**b**) 0–1.4 V at a scan rate of 50 mV·s^−1^.

**Figure 6:**
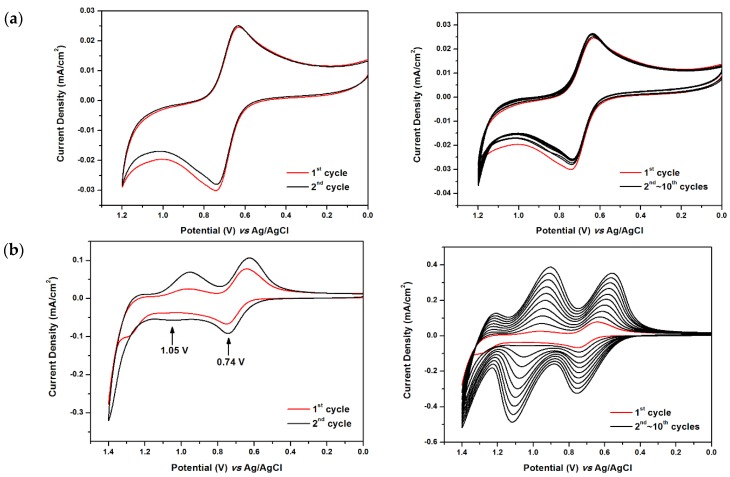
Repetitive cyclic voltammograms of 2 × 10^−4^ M MeOTPA-(NPC)_2_ in 0.1 M Bu_4_NClO_4_/CH_2_Cl_2_ solution in the potential range of (**a**) 0–1.2 V and (**b**) 0–1.4 V at a scan rate of 50 mV·s^−1^.

**Scheme 2:**
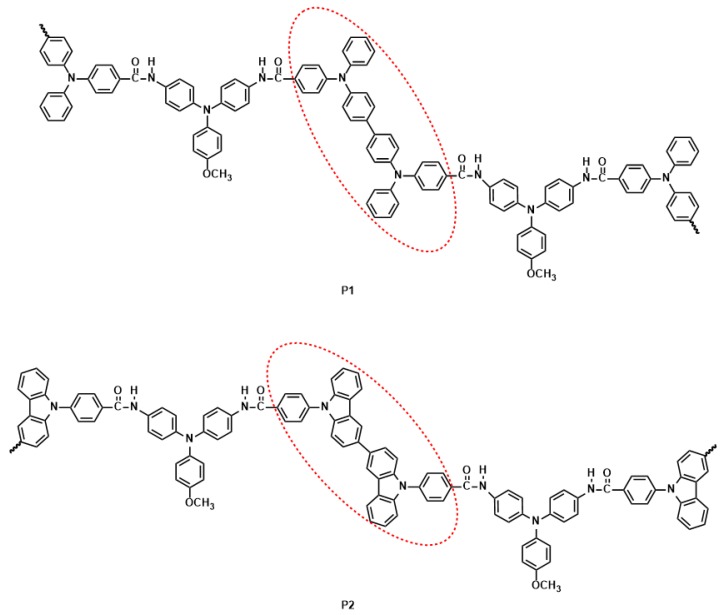
Proposed structures of electro-generated polymers P1 and P2.

Figure 5a presents cyclic voltammetry (CV) scans of 2 × 10^−4^ M MeOTPA-(TPA)_2_ in 0.1 M Bu_4_NClO_4_/CH_2_Cl_2_ at a scan rate of 50 mV·s^−1^ between 0 and 1.2 V. After a ten cycles scan, no new peaks were detected. When the voltage was scanned between 0 and 1.4 V, two oxidation peaks at 0.71 and 1.26 V were observed in the first scan. New oxidation peaks appeared at about 0.95 and 1.08 V in the subsequent scan, indicating the occurrence of the coupling reactions between terminal TPA cationic radicals forming the tetraphenylbenzidine (TPB) segments. Upon successive scanning, the redox wave current intensities gradually increased, which indicates the formation of the electrochemically active polymeric film (coded as P1, Scheme 2) on the electrode surface (Figure 5b). Similarly, the NPC-based monomer MeOTPA-(NPC)_2_ did not undergo electropolymerization when the potential was scanned between 0 and 1.2 V (Figure 6a). As the potential was scanned between 0 and 1.4 V, the carbazole groups started to oxidize and coupling reactions occurred forming the biscarbazole units. As the CV scanning continued, the redox currents intensified (Figure 6b) and a polymer film (coded as P2) gradually deposited on the electrode surface.

**Figure 7:**
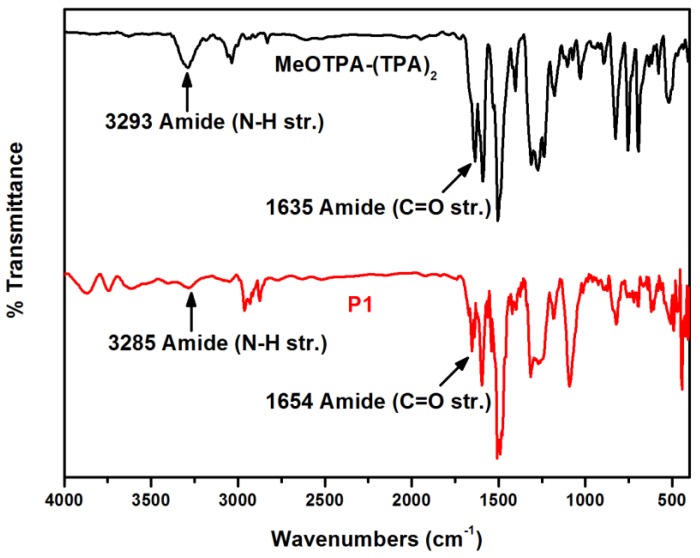
IR spectra of MeOTPA-(TPA)_2_ and P1 film.

As a typical example, the IR spectra of MeOTPA-(TPA)_2_ and its electro-generated P1 film are shown in Figure 7. The P1 film shows the characteristic amide absorption bands at around 3285 cm^−1^ (N–H stretching) and 1654 cm^−1^ (amide C=O stretching). As compared to that of monomers, the P1 film shows very similar IR absorptions in the range of 1200–1700 cm^−1^ being associated with amide C=O, aromatic ring and C−N stretch. The change in the low frequency region 500–1000 cm^−1^, peculiar to benzene ring C–H out-of-plane bending, can be attributed to the TPA coupling reaction.

### 2.4. Optical and Electrochemical Properties of Polymers

The UV-vis absorption spectra of monomers MeOTPA-(TPA)_2_ and MeOTPA-(NPC)_2_ in CH_2_Cl_2_ and their derived polymer films of P1 and P2 on an ITO-glass substrate are shown in Figure S4 (Supplementary Materials). The spectra of the monomers show absorption bands with maximum peaks at 350 and 336 nm and absorption onsets at 408 and 426 nm, respectively. The polymer films of P1 and P2 show absorption maxima at 361 and 338 nm and absorption onsets at 454 and 449 nm, respectively. The red-shift of absorption maximum and onset of the polymer films compared to the monomers implies an extended π-conjugation length.

**Figure 8:**
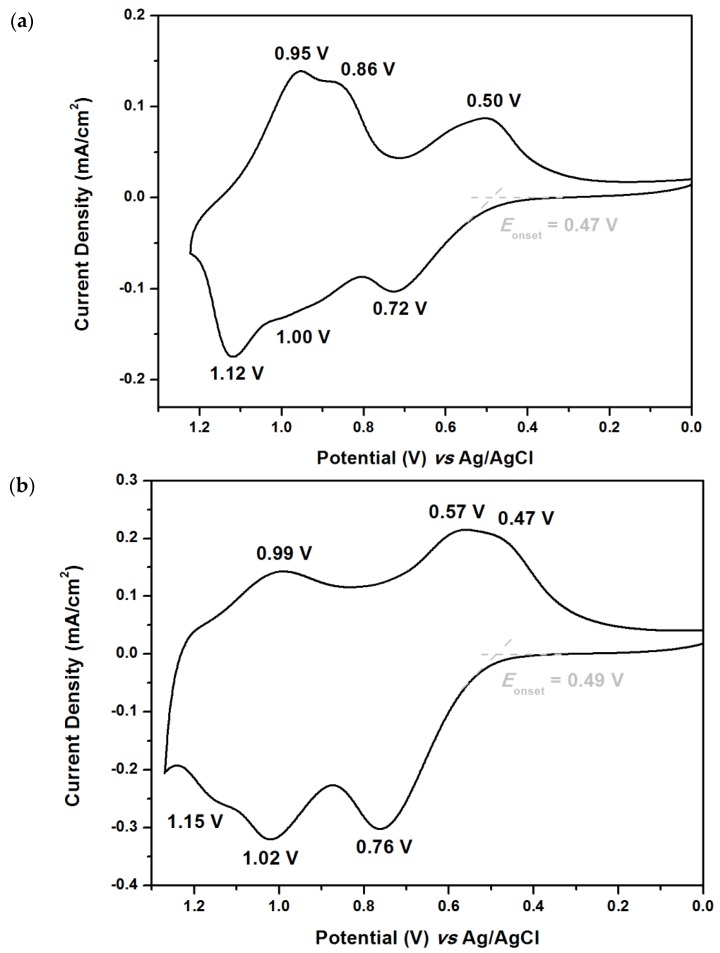
Cyclic voltammograms and differential pulse voltammograms of polymer films (**a**) P1 and (**b**) P2 on the ITO-coated glass slide in 0.1 M Bu_4_NClO_4_/CH_2_Cl_2_ at a scan rate of 50 mV·s^−1^.

The electrochemical behavior of the electro-synthesized polymer films on the ITO-glass substrate were investigated by cyclic voltammetry in a 0.1 M Bu_4_NClO_4_/CH_2_Cl_2_ solution without the monomer. The optical and electrochemical properties of the polymers are summarized in Table 1. As illustrated in Figure 8, the polymer films exhibited three reversible redox couples, at the half-wave potentials (*E*_1/2_) of 0.61, 0.93 and 1.04 V for P1 from MeOTPA-(TPA)_2_, and 0.62, 0.80 and 1.07 V for P2 from MeOTPA-(NPC)_2_. The oxidation onset potential (*E*_onset_) of P1 and P2 were recorded at 0.47 and 0.49 V (vs. Ag/AgCl), respectively. Redox waves at lower potentials of these two polymers are apparently attributable to reversible electrochemical oxidation processes of the MeOTPA center. The energy levels of the highest occupied molecular orbital (HOMO) and lowest unoccupied molecular orbital (LUMO) of the corresponding polymers estimated from the *E*_onset_ values and UV-vis absorption edge are also included in Table 1.

### 2.5. Spectroelectrochemical Properties of Polymers

**Figure 9:**
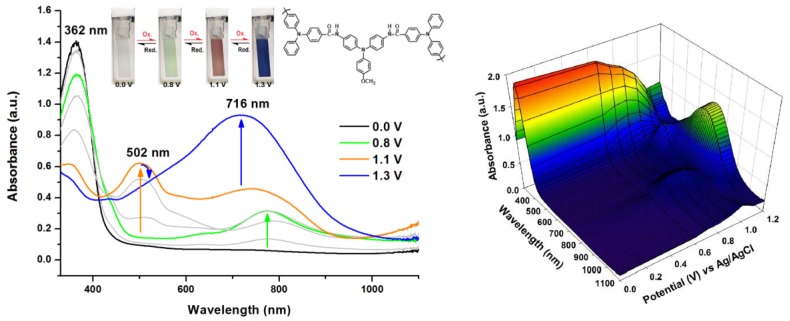
Spectroelectrochemistry of the electro-generated films of P1 (thickness: 250 ± 100 nm) on the ITO-coated glass substrate in 0.1 M Bu_4_NClO_4_/CH_2_Cl_2_ at various applied potentials.

Spectroelectrochemical behaviors of the polymeric films on ITO glass were investigated. Figure 9 illustrates the changes in the electronic absorption spectrum of the P1 film upon changing the applied potentials. The electronic absorption profile of neutral form of the film exhibits absorption maximum at around 362 nm, which is due to π−π* transitions for P1. When the applied potential was gradually raised to 0.8 V, the absorption intensity at 362 nm slightly dropped and new band appeared at 775 nm, and the color changed to pale green (L*: 77; a*: −9; b*: 13), which could be attributed to the formation of MeOTPA radical cation. Upon further oxidation at applied potential to 1.1 V, a new absorption peak at 502 nm was observed and the film changed into an orange color (L*: 50; a*: 17; b*: 15). The spectral change should be originated from the tetraphenylbenzidine radical cation (TPB+•) moieties. When the applied potential was adjusted to 1.3 V, the absorption peak at 502 nm decreased, with a formation of new absorption band at around 716 nm and a color change to blue (L*: 21; a*: 6; b*: −26). This result indicated the formation of TPB dicationic species.

**Figure 10:**
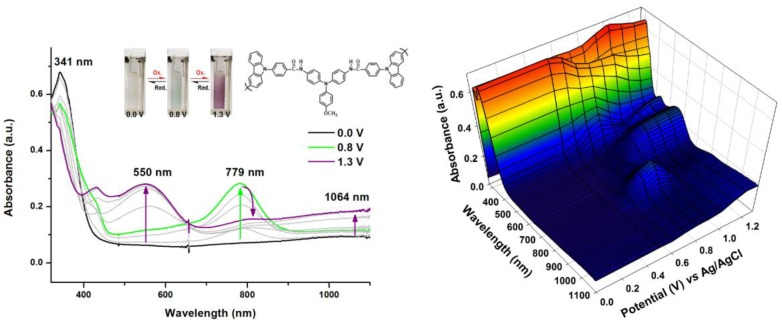
Spectroelectrochemistry of the electro-generated films of P2 (thickness: 170 ± 100 nm) on the ITO-coated glass substrate in 0.1 M Bu_4_NClO_4_/CH_2_Cl_2_ at various applied potentials.

A similar spectral change was observed for the P2 film at the early stage of the oxidation (Figure 10). Upon oxidation at 0.8 V, an absorption band at 341 nm started to decrease and a new band at 779 nm gradually increased in intensity, and the film changed into a pale green color (L*: 65; a*: −8; b*: 7). When the applied voltage was increased to 1.3 V, absorption band at 779 nm decreased in intensity and new absorption bands intensified at 550 nm (L*: 39; a*: 22; b*: −4).

### 2.6. Electrochromic Switching

**Figure 11:**
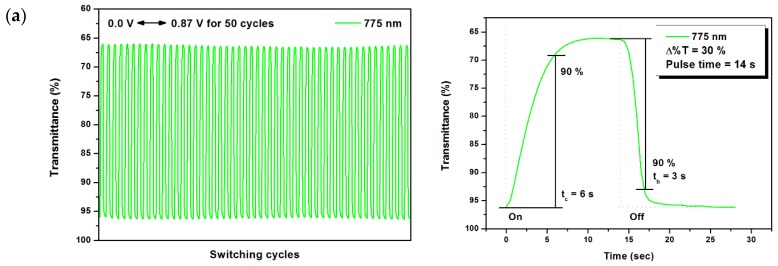

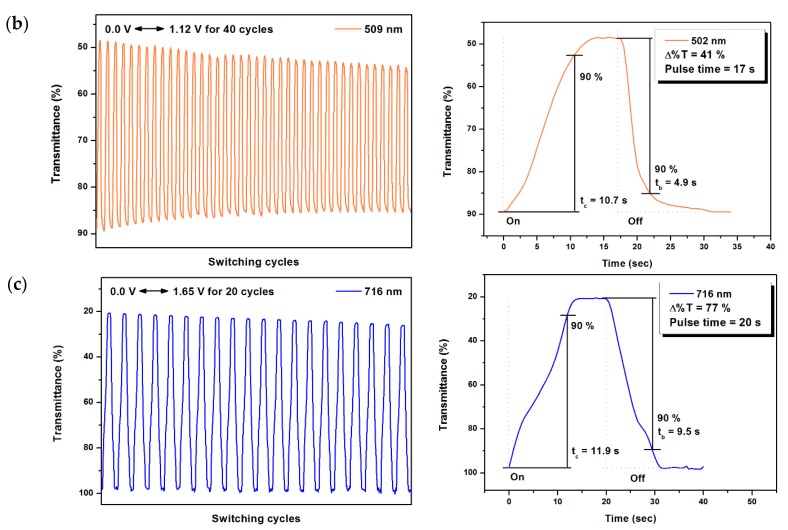
Percentage transmittance changes of the cast film of P1 on the ITO-glass slide (coated area ~1 cm^2^) (in CH_2_Cl_2_ with 0.1 M Bu_4_NClO_4_ as the supporting electrolyte) by applying a potential at (**a**) 0.0 ↹ 0.87 V for 50 cycles, (**b**) 0.0 ↹ 1.12 V for 40 cycles, and (**c**) 0.0 ↹ 1.65 V for 20 cycles.

Double potential step chronoamperometry was carried out to estimate the response time and cycling stability of the electro-generated films. The potential was stepped between neutral and oxidized states of the polymer film with a suitable residence time. During the potential switching, the transmittance percentage (% *T*) at the wavelength of maximum contrast was measured using a UV-vis spectrophotometer. The measurements were conducted using a single wavelength with obvious absorption intensity change. As shown in Figure 11a, for P1 optical contrast (Δ% *T*) and switching time (green coloring, at 90% full-transmittance change) were measured as 30% and 6 s for 775 nm by stepping the potential between 0 and 0.87 V with a residence time of 14 s. As the applied voltage was stepped from 0 to 1.12 V, the film exhibited Δ% *T* of 41% at 509 nm for the oxidized orange state which required 10.7 s for the coloring step and 4.9 s for the bleaching step as shown in Figure 11b. When the applied voltage was switched between 0 and 1.65 V, the film required 11.9 s for the coloring step and 9.5 s for the bleaching step, and Δ% *T* was 77% at 716 nm for the blue coloring (Figure 11c). Coloration efficiency (*CE*) is another important parameter for the electrochromic applications. On the basis of the equation *CE* = Δ*OD*/*Q*_d_, where Δ*OD* is the optical absorbance change, and *Q*_d_ (mC/cm^2^) is the inject/ejected charge during a redox step (Supplementary Materials Figures S5 to S9). The *CE* values of P1 were estimated to be 73 cm^2^/C at 775 nm, 57 cm^2^/C at 509 nm and 65 cm^2^/C at 716 nm (Table 2).

**Figure 12:**
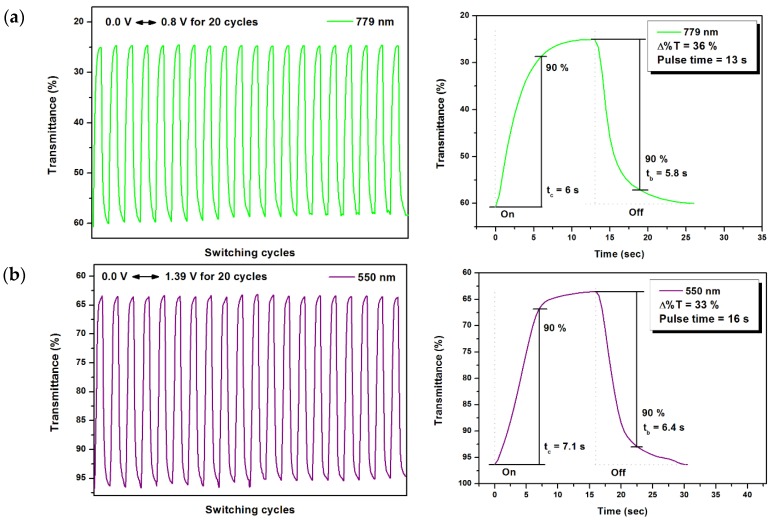
Percentage transmittance changes of the cast films of P2 on the ITO-glass slide (coated area ~1 cm^2^) (in CH_2_Cl_2_ with 0.1 M Bu_4_NClO_4_ as the supporting electrolyte) by applying a potential at (**a**) 0.0 ↹ 0.8 V and (**b**) 0.0 ↹ 1.39 V for 20 cycles.

The switching stability of P2 is illustrated in Figure 12 at 779 and 550 nm with a pulse time of 13 and 16 s, respectively, for 20 cycles. The P2 film exhibited 6 s switching time at 779 nm between its neutral and the first oxidized state. The *CE* value for the green coloring was estimated as 123 cm^2^/C at 779 nm. The switching time and Δ% *T* values for the P2 film were measured as 7.1 s and 33%, respectively, while stepping the potential between 0 and 1.39 V at 550 nm. The high electrochromic switching stability of these two polymers at the first oxidation state could be attributed to their high electrochemical stability of the MeOTPA core.

### 2.7. Electrochromic Devices

**Figure 13:**
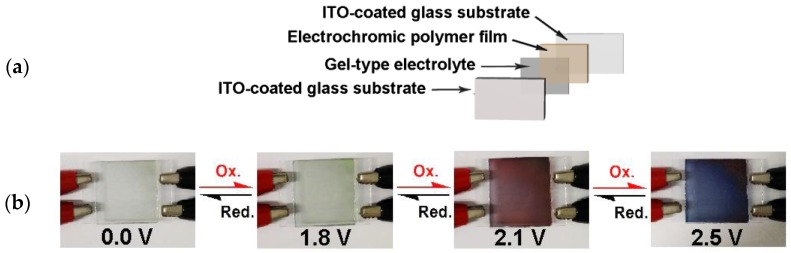
(**a**) Schematic illustration of the structure of the electrochromic devices. (**b**) Photos of the sandwich-type ITO-coated glass electrochromic device using P1 film as active layer at indicated applied voltages.

Based on the above-mentioned results, it can be concluded that the electro-generated polymers may find use in the construction of electrochromic devices (ECD) and optical display. Therefore, we fabricated single layer electrochromic cells as preliminary investigations. The schematic illustration of the structure of the ECD is shown in Figure 13a. The ECD using P1 as active layer was fabricated (Figure 13b). The ECD is colorless in its neutral state, and it slowly switched to pale green, maroon, and blue color at an increasing oxidation voltage of 1.8, 2.1, and 2.5 V, respectively.

## 3. Conclusions

Two triphenylamine-based compounds MeOTPA-(TPA)**_2_** and MeOTPA-(NPC)**_2_** composed of a central MeOTPA core connected through amide linkers to terminal triphenylamine or *N*-phenylcarbazole units were developed as electroactive monomers to prepare electrochromic poly(amide-amine) films on the ITO electrode surface through electropolymerization. The electro-generated polymer films exhibited reversible redox processes and multi-colored electrochromic behaviors upon electro-oxidation. In general, the electrochromic films possessed moderate coloration efficiency and good cycling stability. The electrochemically synthesized polymers might be promising candidates for electrochromic materials.

## Figures and Tables

**Table 1:** Optical and electrochemical properties of the electrochemically synthesized polymers.

Polymers	UV-Vis Absorption (nm) ^a^		Oxidation Potential (V) ^b^				*E*_g_ (eV) ^c^	HOMO (eV) ^d^	LUMO (eV) ^d^
	λ_max_	λ_onset_	*E*_onset_	*E*_1/2_^Ox1^	*E*_1/2_^Ox2^	*E*_1/2_^Ox3^			
P1	361	454	0.47	0.61	0.93	1.04	2.73	4.83	2.10
P2	338	449	0.49	0.62	0.80	1.07	2.76	4.85	2.09

^a^ UV-vis absorption maximum and onset wavelengths for the polymer films. ^b^ Calculated from first cyclic voltammetry (CV) scans, versus Ag/AgCl in CH_2_Cl_2_ at a scan rate of 50 mV·s^–1^. ^c^ Optical bandgap calculated from absorption edge of the polymer film: *E*_g_ = 1240/λ_onset_. ^d^ The highest occupied molecular orbital (HOMO) and lowest unoccupied molecular orbital (LUMO) energy levels were calculated from *E*_onset_ values of CV diagrams and were referenced to ferrocene (4.8 eV relative to vacuum energy level; *E*_onset_ = 0.44 V; *E*_1/2_ = 0.52 V in CH_2_Cl_2_). EHOMO = *E*_onset_ + 4.8 − 0.44 (eV); ELUMO = EHOMO − *E*g.

**Table 2:** Electrochromic properties of the polymer films.

Polymers	λ_max_^a^ (nm)	Δ% *T*	Response Time ^b^		Δ*OD* ^c^	*Q*_d_^d^ (mC/cm^2^)	*CE* ^e^ (cm^2^/C)
			*t*_c_ (s)	*t*_b_ (s)			
P1	775	30	6.0	3.0	0.16	2.21	73
	502	41	10.7	4.9	0.26	4.56	57
	716	77	11.9	9.5	0.68	10.39	65
P2	779	36	6.0	5.8	0.38	3.09	123
	550	33	7.1	6.4	0.18	4.25	42

^a^ Wavelength of absorption maximum. ^b^ Time for 90% of the full-transmittance change. ^c^ Optical density change (Δ*OD*) = log (*T*_bleached_/*T*_colored_), where *T*_colored_ and *T*_bleached_ are the maximum transmittance in the oxidized and neutral states, respectively. ^d^ *Q*_d_ is ejected charge, determined from the in situ experiments. ^e^ Coloration efficiency (*CE*) = Δ*OD*/*Q*_d_.

## Supplementary Materials

Synthetic details and characterization data of the synthesized compounds are available online at www.mdpi.com/2073-4360/9/12/708/s1.
