# Supplementary material for: Electrosynthesis of Aromatic Poly(amide-amine) Films from Triphenylamine-Based Electroactive Compounds for Electrochromic Applications
Source: Polymers (Basel). 2017 Dec 13;9(12):708. doi: 10.3390/polym9120708 (PMC6418588; doi:10.3390/polym9120708)
Supplement: Supplementary file 1 [file polymers-09-00708-s001.pdf]

Supplementary Materials

Electrosynthesis of Aromatic Poly(amide-amine) Films from Triphenylamine-based Electroactive Compounds for Electrochromic Applications

Sheng-Huei Hsiao*, Hsing-Yi Lu

Department of Chemical Engineering and Biotechnology, National Taipei University of Technology, No. 1, Sec. 3, Chunghsiao East Rd., Taipei 10608, Taiwan; shinhwa904@gmail.com

* Correspondence: shhsiao@ntut.edu.tw

Materials

p-Anisidine (Acros, Geel, Belgium), *p*-fluoronitrobenzene (Acros, Geel, Belgium), cesium fluoride (CsF, Acros, Geel, Belgium), hydrazine monohydrate (Wako, Osaka, Japan), 10% palladium on activated carbon (Pd/C, Fluka, Milwaukee, WI, USA), dimethyl sulfoxide (DMSO, Tedia, Fairfield, OH, USA), pyridine (Py, Wako, Osaka, Japan), *N*-methyl-2-pyrrolidone (NMP, Tedia, Fairfield, OH, USA), and other reagents and solvents were used as received from commercial sources. According to a reported synthetic procedure [S1], 4,4′-diamino-4″-methoxytriphenylamine (**MeOTPA-(NH$_2$)$_2$**) was prepared by the fluoro-displacement of *p*-fluoronitrobenzene with *p*-anisidine in the presence of CsF in DMSO, followed by Pd/C-catalyzed hydrazine reduction of the intermediate dinitro compound 4-methoxy-4′,4″-dinitrotriphenylamine (**MeOTPA-(NO$_2$)$_2$**) in ethanol (Scheme 1 in the main text). As described previously [S2], 4-carboxytriphenylamine (**TPA-COOH**) and *N*-(4-carboxyphenyl)carbazole (**NPC-COOH**) were readily prepared from *N*-arylation reactions of diphenylamine and carbazole, respectively, with *p*-fluorobenzonitrile, followed by alkaline hydrolysis of the intermediate cyano compounds.

4.,4′-Bis(4-Diphenylaminobenzamido)-4″-Methoxy-Triphenylamine (MeOTPA-(TPA)$_2$)

A mixture of 0.3 g (1 mmol) of **MeOTPA-(NH$_2$)$_2$**, 0.64 g (2.2 mmol) of 4-carboxytriphenylamine (**TPA-COOH**), 0.6 mL of triphenyl phosphite (TPP), 0.2 mL of pyridine, and 1 mL of NMP in a 50 mL round-bottom flask equipped was heated with stirring at 120 °C for 3 h. The solution was poured slowly into 150 mL methanol. The precipitated product was collected by filtration, washed repeatedly with methanol, and dried to give 0.7474 g (88%) of the desired monomer **MeOTPA-(TPA)$_2$** as yellow green powder.

IR (KBr): 1635 cm^{-1} (amide C=O stretch), 3293 cm^{-1} (amide N−H stretch). ^{1}H NMR (600 MHz, DMSO-d_6, δ, ppm): 3.74 (s, 3H, −OCH$_3$), 6.90–6.92 (two overlapped doublets, 6H, H$_b$ + H$_f$), 6.96 (d, *J* = 8.8 Hz, 4H, H$_c$), 6.99 (d, *J* = 8.9 Hz, 2H, H$_a$), 7.10 (d, *J* = 7.6 Hz, 8H, H$_g$), 7.14 (t, *J* = 7.4 Hz, 4H, H$_i$), 7.37 (t, *J* = 7.8 Hz, 8H, H$_h$), 7.63 (d, *J* = 8.9 Hz, 4H, H$_d$), 7.84 (d, *J* = 8.8 Hz, 4H,

H_e), 9.99 (s, 2H, amide N−H). FAB-MS: m/z = 847.3522 (M^+); calculated exact mass for $C_{57}H_{45}N_5O_3$ = 847.3533.

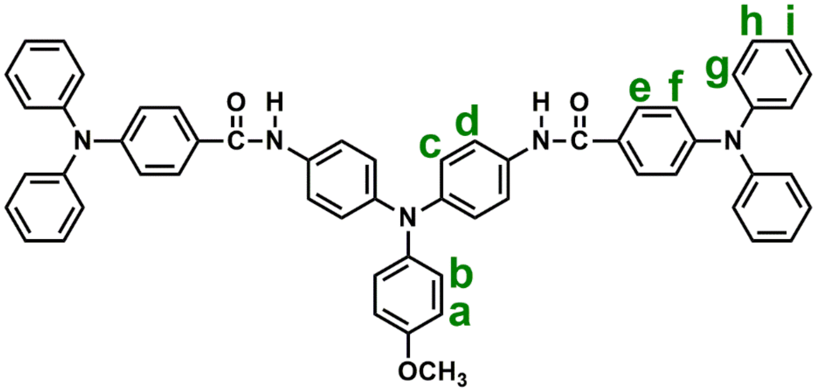

Structure 1. Structure of **MeOTPA-(TPA)$_2$** and code of hydrogen atoms.

4.,4′-Bis[4-(Carbazol-9-Yl)Benzamido]-4′′-Methoxy-Triphenylamine (MeOTPA-(NPC)$_2$)

In a 50 mL round-bottom flask equipped with a stirring bar, a mixture of 0.3 g (1 mmol) of **MeOTPA-(NH$_2$)$_2$**, 0.632 g (2.2 mmol) of *N*-(4-carboxyphenyl)carbazole (**NPC-COOH**), 0.6 mL of TPP, 0.2 mL pyridine, and 2 mL of NMP was heated with stirring at 120 °C for 3 h. The solution was poured slowly into 150 mL methanol. The precipitated product was collected by filtration, washed repeatedly with methanol, and dried to yield 0.7858 g (93%) of a pale yellow powder.

IR (KBr): 1650 cm^{-1} (amide C=O stretch), 3267 cm^{-1} (amide N−H stretch). ^1H NMR (600 MHz, DMSO-d_6, δ, ppm): 3.77 (s, 3H, −OCH_3), 6.95 (d, *J* = 8.9 Hz, 2H, H_b), 7.00 (d, *J* = 8.8 Hz, 4H, H_c), 7.05 (d, *J* = 8.9 Hz, 2H, H_a), 7.33 (t, *J* = 7.8 Hz, 4H, H_i),7.46–7.49 (m, 8H, H_g + H_h), 7.75 (d, *J* = 8.9 Hz, 4H, H_d), 7.82 (d, *J* = 8.4 Hz, 4H, H_f), 8.26–8.28 (two overlapped doublets, 8H, H_j + H_e), 10.38 (s, 2H, amide N−H). ESI-MS: m/z = 843.3209 (M^+); calculated exact mass for $C_{57}H_{41}N_5O_3$ = 843.3195.

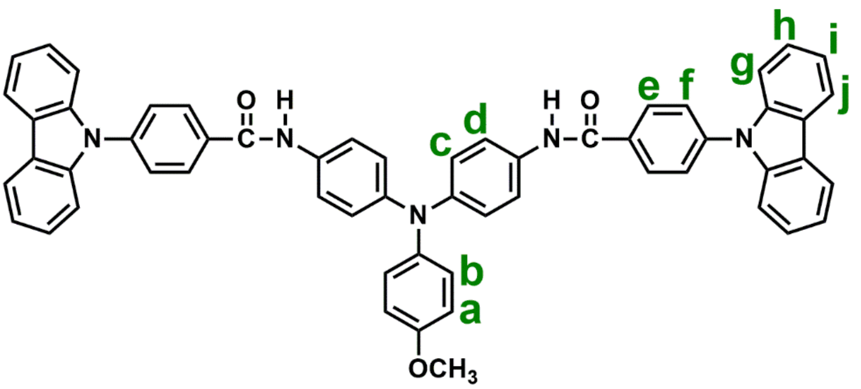

Structure 2. Structure of **MeOTPA-(NPC)$_2$** and code of hydrogen atoms.

Synthesis of Model Compound

In a 50 mL round-bottom flask equipped with a stirring bar, a mixture of 0.3 g (1 mmol) of **MeOTPA-(NH$_2$)$_2$**, 0.269 g (2.2 mmol) of benzoic acid, 0.6 mL of TPP, 0.2 mL pyridine, and 1 mL of NMP was heated with stirring at 120 °C for 3 h. The solution was poured slowly into 150 mL methanol. The precipitated product was collected by filtration, washed thoroughly with methanol, and dried to give 0.187 g (36%) of the model compound, 4,4′-dibenzamido-4″-methoxytriphenylamine (**MeOTPA-(Ph)$_2$**), as pale yellow powder.

IR (KBr): 1649 cm^{-1} (amide C=O stretch), 3279 cm^{-1} (amide N−H stretch). ^{1}H NMR (600 MHz, DMSO-d_6, δ, ppm): 3.75 (s, 3H, −OCH$_3$), 6.92 (d, J = 9.0 Hz, 2H, H$_b$), 6.94 (d, J = 8.9 Hz, 2H, H$_c$), 7.01 (d, J = 8.9 Hz, 2H, H$_a$), 7.52 (t, J = 7.8 Hz, 4H, H$_f$), 7.58 (t, J = 7.3 Hz, 2H, H$_g$), 7.67 (d, J = 9.0 Hz, 4H, H$_d$), 7.94 (d, J = 7.1 Hz, 4H, H$_e$), 10.18 (s, 2H, amide N−H).

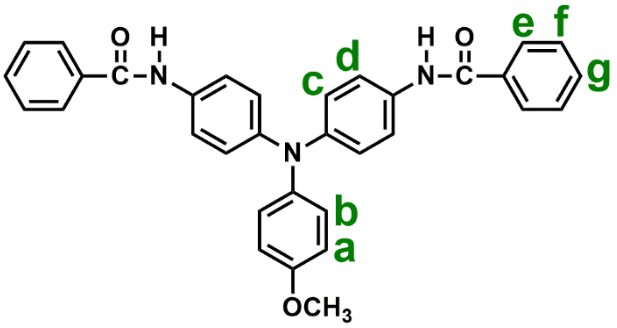

Structure 3. Structure of **MeOTPA-(Ph)$_2$** and code of hydrogen atoms.

Instrumentation

Infrared (IR) spectra were recorded on a Horiba FT-720 FT-IR spectrometer (Kyoto, Japan). ^{1}H NMR spectra was measured on a Bruker Avance III HD-600 MHz NMR spectrometer (Billerica, MA, USA) with tetramethylsilane (TMS) as an internal standard. HRMS were obtained on a JEOL JMS-700 mass spectrometer (Peabody, MA, USA) with ESI resource. Electrochemical measurements were performed with a CH Instruments 750A electrochemical analyzer (Austin, TX, USA). The polymers were electropolymerized from 2 × 10^{-4} M monomers in 0.1 M Bu$_4$NClO$_4$-dichloromethane solution via repetitive cycling at a scan rate of 50 mV/s. Voltammograms are presented with the positive potential pointing to the left and with increasing anodic currents pointing downwards. Cyclic voltammetry was conducted with the use of a three-electrode cell in which indium tin oxide (ITO) (polymer films area about 0.8 cm × 2.4 cm) was used as a working electrode. A platinum wire used as an auxiliary electrode. All cell potentials were taken with the use of a home-made Ag/AgCl, KCl (sat.) reference electrode. Ferrocene was used as an external reference for calibration (+ 0.48 V vs. Ag/AgCl). Spectroelectrochemistry analyses were carried out with an electrolytic cell, which was composed of a 1 cm cuvette, ITO as a working electrode, a platinum wire as an auxiliary electrode, and Ag/AgCl reference electrode. Spectroelectrochemical experiments of model compounds were performed with an optically transparent thin-layer

electrochemical (OTTLE) cell equipped with a Pt minigrid working electrode. Absorption spectra in the spectroelectrochemical experiments were measured with an Agilent 8453 UV-Visible diode array spectrophotometer. Color coordinates of the electrochromic films were measured on an Admesy Brontes colorimeter. Color coordinate measurements were performed for the polymer films at an observational angle of 45° and a standard D65 light source with the Commission internationale de l'Eclairage (CIE) 1976 L* a* b* Color Space.

Electrochemical Polymerization

Electrochemical polymerization was performed with a CH Instruments 750A electrochemical analyzer (Austin, Texas, USA). The polymers were synthesized from 2×10^{-4} M monomers in a dichloromethane (CH_2Cl_2) solution containing 0.1 M Bu_4NClO_4 via repetitive cycling between 0 and 1.4 V at a scan rate of 50 mV/s for ten cycles. The polymer was deposited onto the surface of the working electrode (ITO/glass surface, polymer films area about 0.8 cm × 2.4 cm), and the film was rinsed with plenty of acetone for the removal of un-reacted monomer, inorganic salts and other organic impurities formed during the process.

Fabrication of Electrochromic Devices

Electrochromic polymer films were electrodeposited on the ITO-coated glass substrate by the electropolymerization method described above. A gel electrolyte based on PMMA (Mw: 120000) and $LiClO_4$ was plasticized with propylene carbonate to form a highly transparent and conductive gel. PMMA (1 g) was dissolved in dry CH_2Cl_2 (4 mL), and $LiClO_4$ (0.1 g) was added to the polymer solution as supporting electrolyte. Then propylene carbonate (1.5 g) was added as plasticizer. The mixture was then gently heated until gelation. The gel electrolyte was spread on the polymer-coated side of the electrode, and the electrodes were sandwiched. Finally, an epoxy resin was used to seal the device.

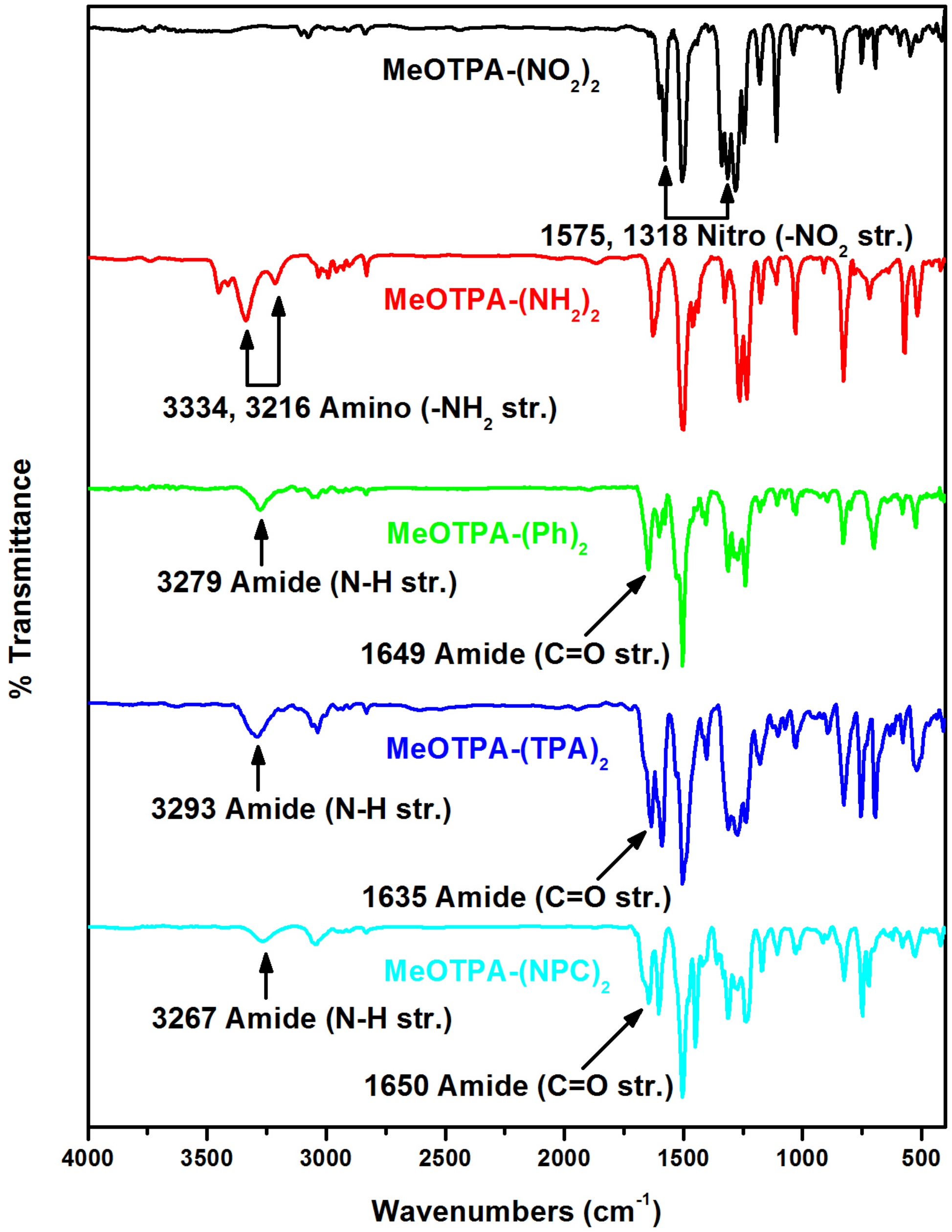

Figure S1. IR spectra of intermediate dinitro compound, diamino compound, model compound, and the target monomers.

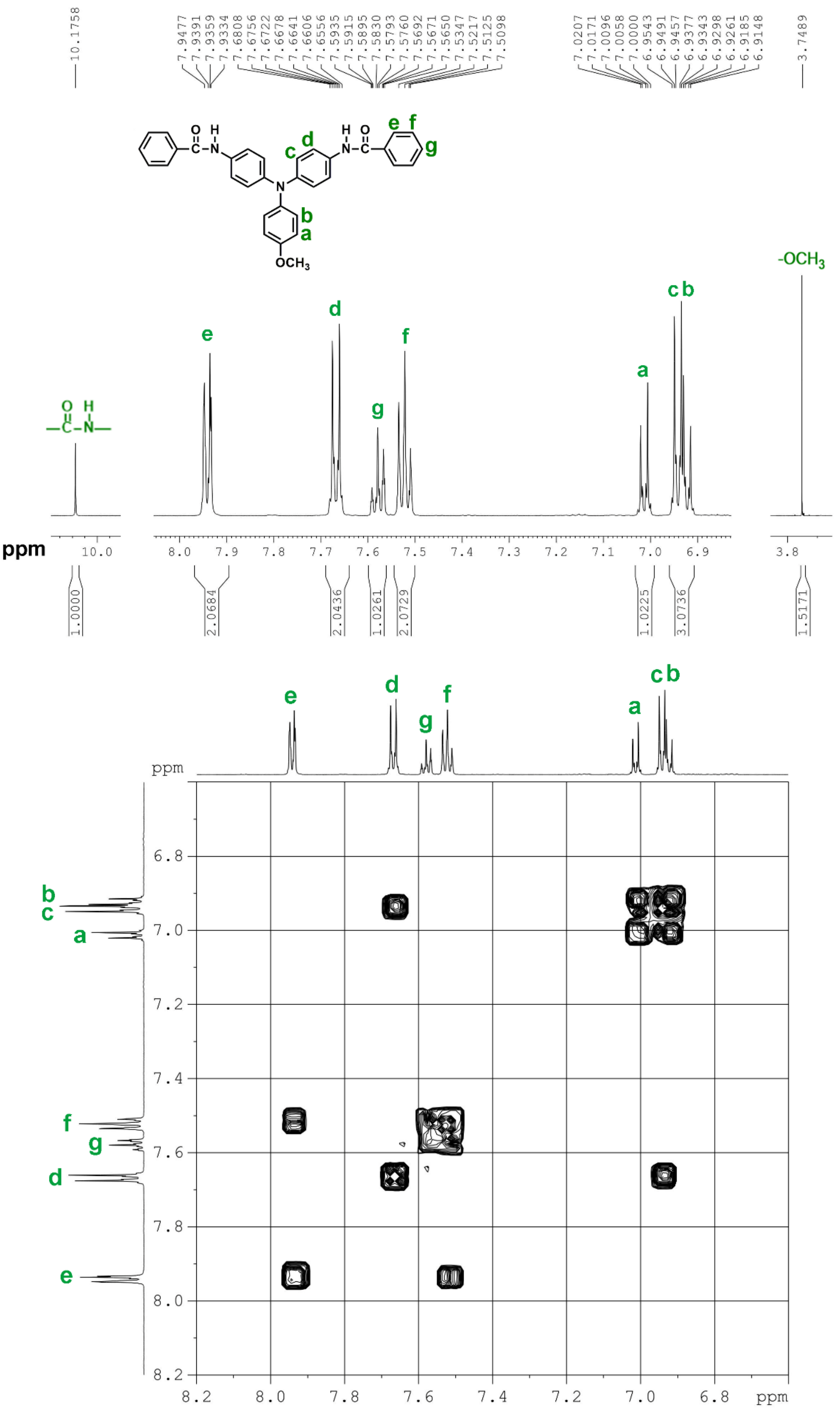

Figure S2. ^{1}H NMR and H-H Correlation Spectroscopy (COSY) spectra of MeOTPA-$(Ph)_2$ in DMSO-d_6.

(a)

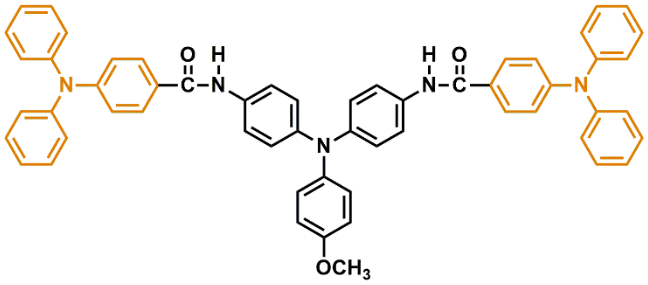

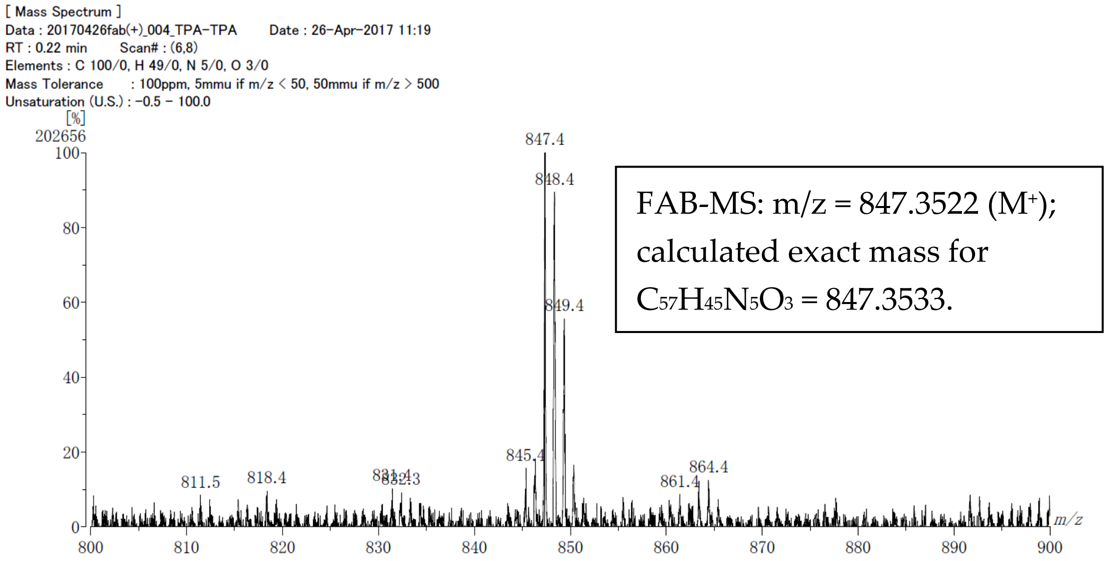

FAB-MS: m/z = 847.3522 (M+);
calculated exact mass for
$C_{57}H_{45}N_5O_3$ = 847.3533.

(b)

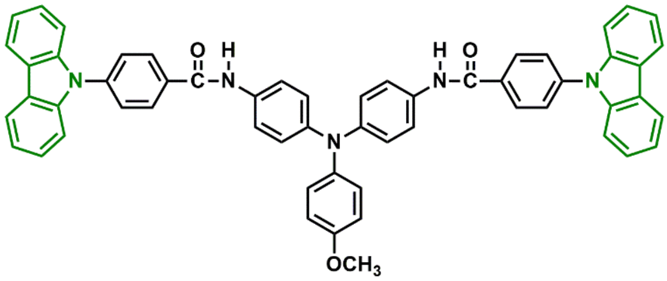

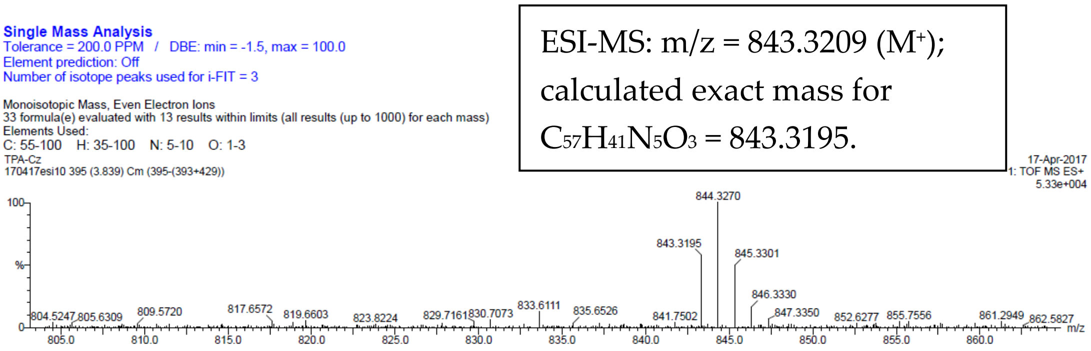

ESI-MS: m/z = 843.3209 (M+);
calculated exact mass for
$C_{57}H_{41}N_5O_3$ = 843.3195.

Figure S3. Mass spectra of (**a**) MeOTPA-(TPA)$_2$ and (**b**) MeOTPA-(NPC)$_2$.

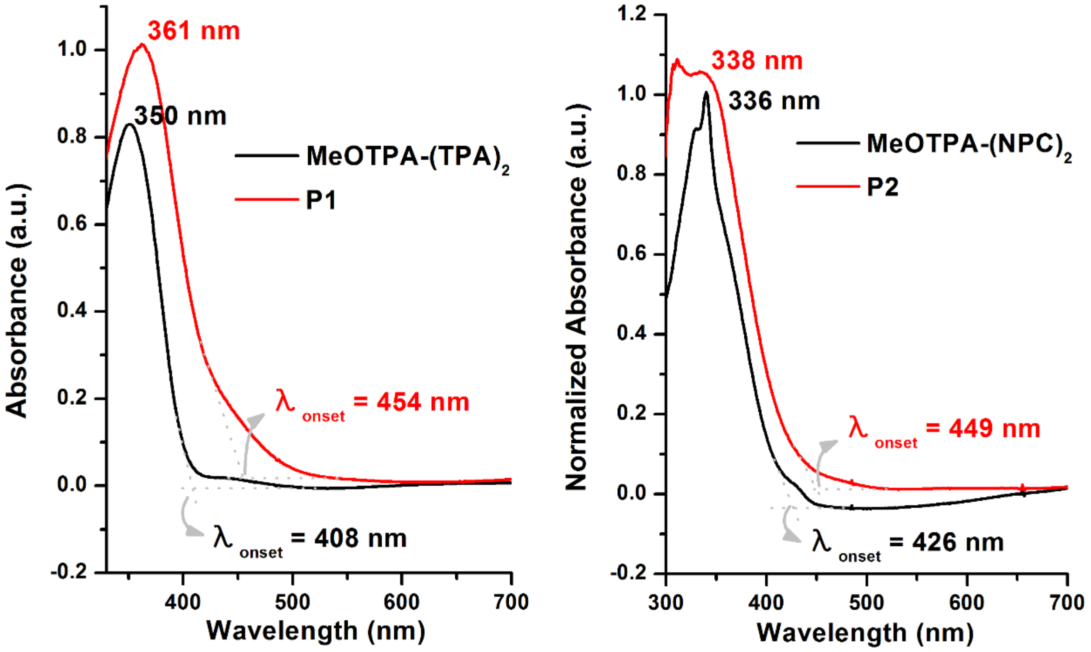

Figure S4. UV-vis absorption spectra of MeOTPA-(TPA)$_2$ and MeOTPA-(NPC)$_2$ in CH_2Cl_2 and the deposited polymer films on ITO-glass.

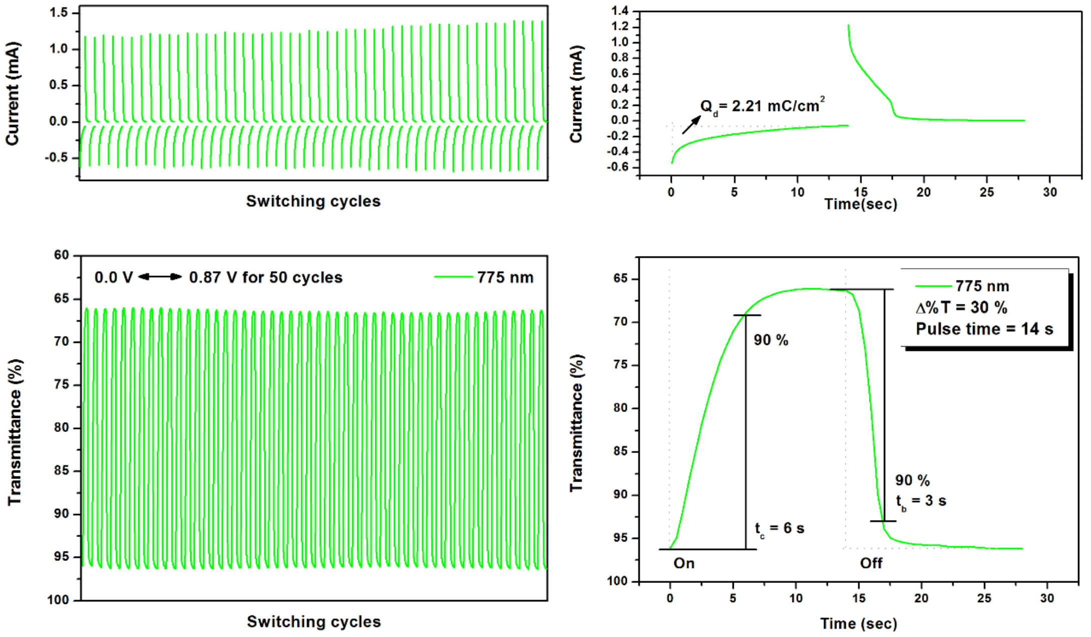

Figure S5. Potential step absorptiometry of the cast films of **P1** on the ITO-glass slide (coated area ~ 1 cm^2) (in CH_2Cl_2 with 0.1 M Bu_4NClO_4 as the supporting electrolyte) by applying a potential at 0.0 ⇆0.87 V for 50 cycles.

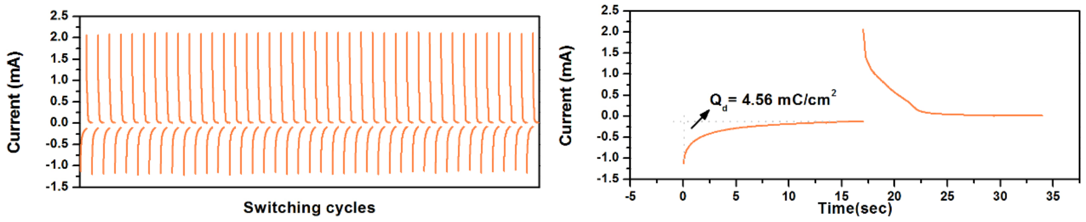

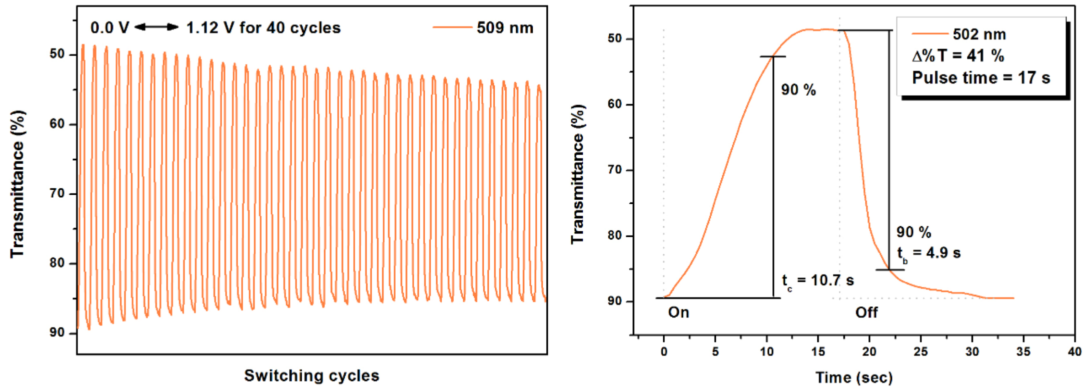

Figure S6. Potential step absorptiometry of the cast films of **P1** on the ITO-glass slide (coated area ~ 1 cm^2) (in CH_2Cl_2 with 0.1 M Bu_4NClO_4 as the supporting electrolyte) by applying a potential at 0.0 ⇆1.12 V for 40 cycles.

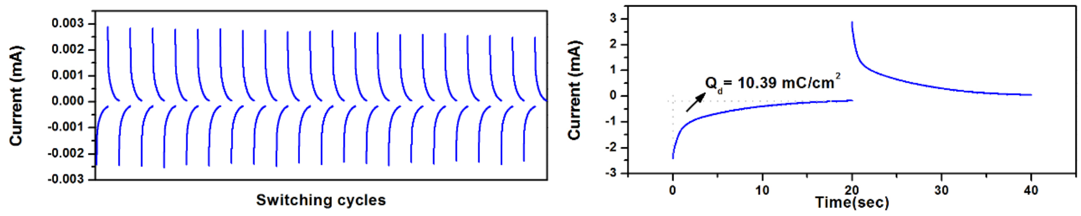

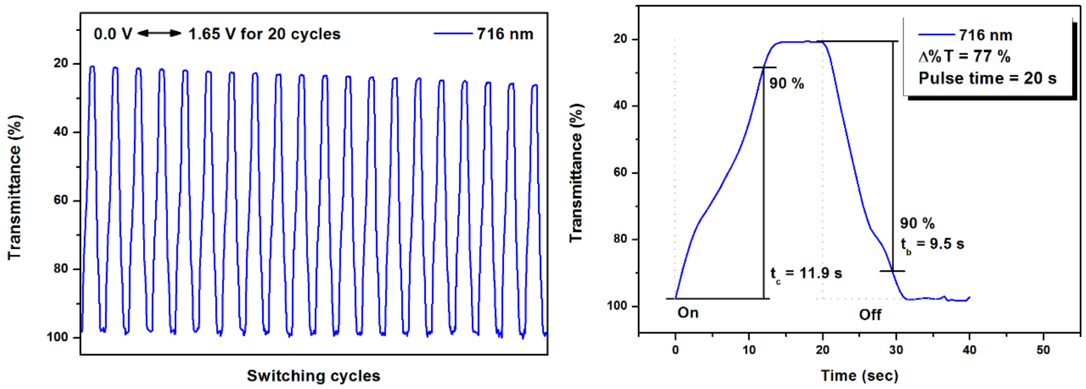

Figure S7. Potential step absorptiometry of the cast films of **P1** on the ITO-glass slide (coated area ~ 1 cm^2) (in CH_2Cl_2 with 0.1 M Bu_4NClO_4 as the supporting electrolyte) by applying a potential at 0.0 ⇆1.65 V for 20 cycles.

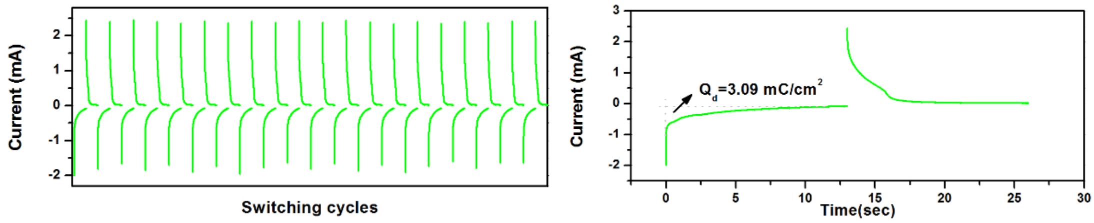

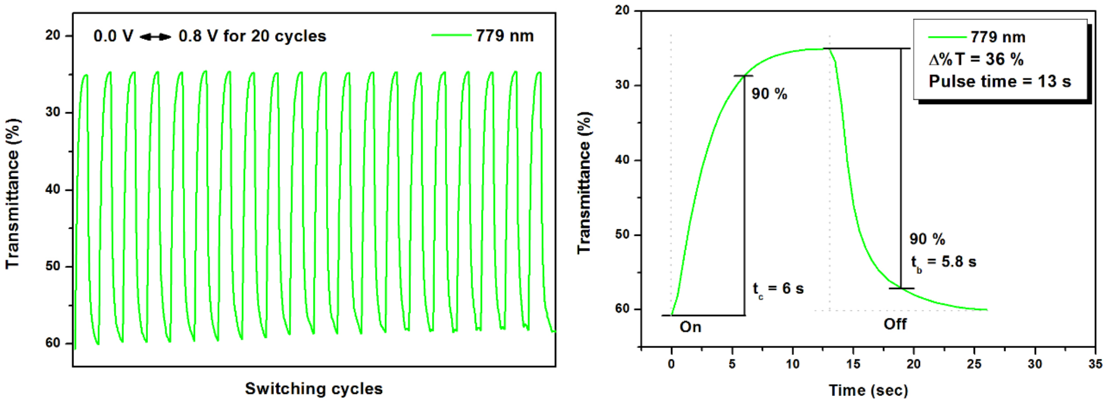

Figure S8. Potential step absorptiometry of the cast films of **P2** on the ITO-glass slide (coated area ~ 1 cm^2) (in CH_2Cl_2 with 0.1 M Bu_4NClO_4 as the supporting electrolyte) by applying a potential at 0.0 ⇆0.8 V for 20 cycles.

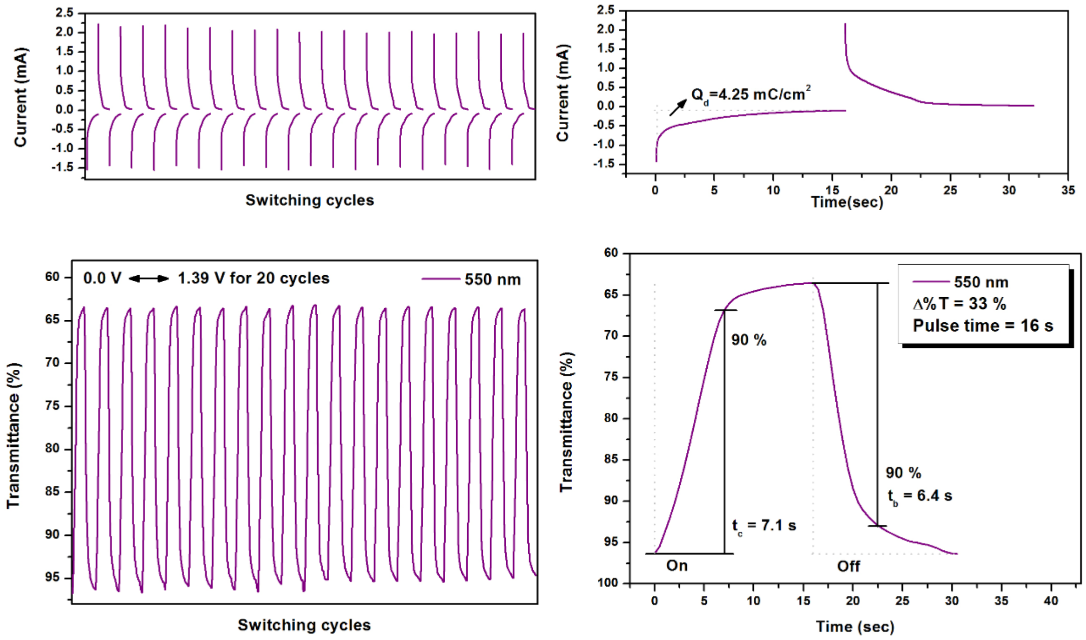

Figure S9. Potential step absorptiometry of the cast films of **P2** on the ITO-glass slide (coated area ~ 1 cm^2) (in CH_2Cl_2 with 0.1 M Bu_4NClO_4 as the supporting electrolyte) by applying a potential at 0.0 ⇆1.39 V for 20 cycles.

References

S1. Chang, C.-W.; Liou, G.-S.; Hsiao, S.-H. Highly stable anodic green electrochromic aromatic polyamides: Synthesis and electrochromic properties, *J. Mater. Chem.* 2007, *17*, 1007–1015.

S2. Hsiao, S.-H.; Lin, J.-Y. Electrosynthesis of ambipolar electrochromic polymer films from anthraquinone-triarylamine hybrids. *J. Polym. Sci. A* **2016**, *54*, 644−655.
